# Endoscopic gastrointestinal bypass anastomosis using deformable self-assembled magnetic anastomosis rings (DSAMARs) in a pig model

**DOI:** 10.1186/s12876-024-03122-0

**Published:** 2024-01-05

**Authors:** Miaomiao Zhang, Jianqi Mao, Jia Ma, Shuqin Xu, Yi Lyu, Xiaopeng Yan

**Affiliations:** 1 Department of Hepatobiliary Surgery, The First Affiliated Hospital of Xi’an Jiaotong University, 710061 Xi’an, China; 2 National and Local Joint Engineering Research Center of Precision Surgery & Regenerative Medicine, The First Affiliated Hospital of Xi’an Jiaotong University, 710061 Xi’an, China; 3 Zonglian College, Xi’an Jiaotong University, 710061 Xi’an, China; 4 Department of Surgical Oncology, Shaanxi Provincial People’s Hospital, 710068 Xi’an, China

**Keywords:** Gastroscope, Magnetic surgery, Magnetic compression technique, Magnamosis, Gastrointestinal bypass anastomosis

## Abstract

**Background:**

To investigate the feasibility of a deformable self-assembled magnetic anastomosis ring (DSAMAR), designed and developed by us, for endoscopic gastrointestinal bypass anastomosis.

**Methods:**

Ten experimental pigs were used as model animals. The DSAMAR comprises 10 trapezoidal magnetic units, arranged in a straight line under the constraint of a guide wire. When the desired anastomosis site is reached under the guidance of an endoscope, the catheter pushes the magnetic unit along the guide wire. The linear DSAMAR can be assembled into a circular DSAMAR. Two DSAMARs were inserted, one at the end of the duodenum and the other into the stomach successively. They attracted each other and compressed the wall of the stomach and duodenum to establish gastrointestinal bypass anastomosis. The experimental pigs were euthanized 4 weeks after the operation, and the gastrointestinal bypass anastomosis specimens were obtained. The anastomosis formation was evaluated by the naked eye and histology.

**Results:**

Gastrointestinal bypass anastomosis with DSAMARs was successfully performed. The average operation time under an endoscope was 70.30 ± 19.05 min (range: 43–95 min). The DSAMARs were discharged through the anus 10–17 days after surgery. There were no complications such as gastrointestinal bleeding, perforation, anastomotic fistula, and gastrointestinal obstruction during and after the operation. Gastroscopy and gross specimen of the anastomosis showed a well-formed magnetic anastomosis. Histological observation showed good continuity of the serous membrane and the mucosa of magnetic anastomosis.

**Conclusion:**

The DSAMAR is a safe and feasible device for fashioning gastrointestinal bypass anastomosis in this animal model.

**Supplementary Information:**

The online version contains supplementary material available at 10.1186/s12876-024-03122-0.

## Background

Gastric outlet obstruction, often caused by pancreatic cancer and gastric adenoma, is a common adverse event in malignant tumors of the digestive system [1]. Patients with gastric outlet obstruction are unable to eat through the mouth, which in serious cases causes severe wasting and malnutrition, greatly reducing the quality of life. For patients who cannot undergo radical tumor surgery, the main therapeutic goals are to relieve obstruction, resume oral feeding, and improve malnutrition. The clinically available methods include endoscopic intestinal stent implantation [2, 3], gastrointestinal bypass surgery, and endoscopic gastrointestinal bypass anastomosis [4]. However, the risk of invasive surgery, whether open or laparoscopic, for these special populations cannot be ignored. Therefore, endoscopic treatment has emerged as a less invasive alternative to surgical gastrojejunostomy for palliating the symptoms of malignant gastric outlet obstruction (MGOO) [5, 6].

However, endoscopic gastrointestinal stent placement can be effective only in some patients with gastric outlet stenosis. For example, complete obstruction of the lumen owing to the tumor or re-stenosis owing to continued tumor growth after stent implantation cannot be corrected by endoscopic stenting. Other patients may need endoscopic gastrointestinal bypass anastomosis. With the maturity of EUS technology and the development of metal stents for application in the gastrointestinal cavity, endoscopic gastrointestinal bypass anastomosis has become relatively common in clinical practice and has yielded good clinical results. However, it is difficult to operate and has high technical requirements for endoscopists; therefore, it can only be carried out at the endoscopy centers of a few hospitals with extensive therapeutic EUS experience [7].

In 1995, Cope combined endoscopy with magnetic compression anastomosis [8], creating a new model for endoscopic gastrointestinal anastomosis. However, the magnet needs to be flexible for successful endoscopic gastrointestinal magnetic anastomosis. Magnets with diverse designs have been developed for application in endoscopic gastrointestinal anastomosis, varying from the initially developed round magnets to oval magnets [9, 10] and to the currently used deformable magnets [11–13], reflecting the continuous improvement and optimization of research on endoscopic magnetic anastomosis. The magnet used for the endoscopic treatment of gastric outlet obstruction must be able to pass through a narrow lumen and establish a larger anastomosis. To this end, we designed a deformable self-assembly magnetic anastomosis ring (DSAMAR). This study investigates the feasibility of DSAMARs for endoscopic gastrointestinal bypass anastomosis using pigs as a model.

## Methods

### Ethical statement

The experimental protocol was reviewed and approved by the Committee for Ethics of Animal Experiments of Xi’an Jiaotong University (number 2022 − 1451). And the study was reported in accordance with ARRIVE guidelines. Ten Qinchuan white pigs (males = 5, females = 5) with body weights of 20–25 kg were obtained from the Experimental Animal Center of Xi’an Jiaotong University.

### Study design

This study was a feasibility study. Therefore, no control group was set up. All 10 pigs were included in the experimental group. DSAMARs were used to complete gastrointestinal bypass magnetic anastomosis in all experimental pigs under endoscopic operation. The endoscopic operation time, complications, and magnet-discharge time were recorded. The experimental pigs were euthanized 4 weeks after the operation to evaluate the formation of anastomosis.

### DSAMAR

**Fig. 1:**
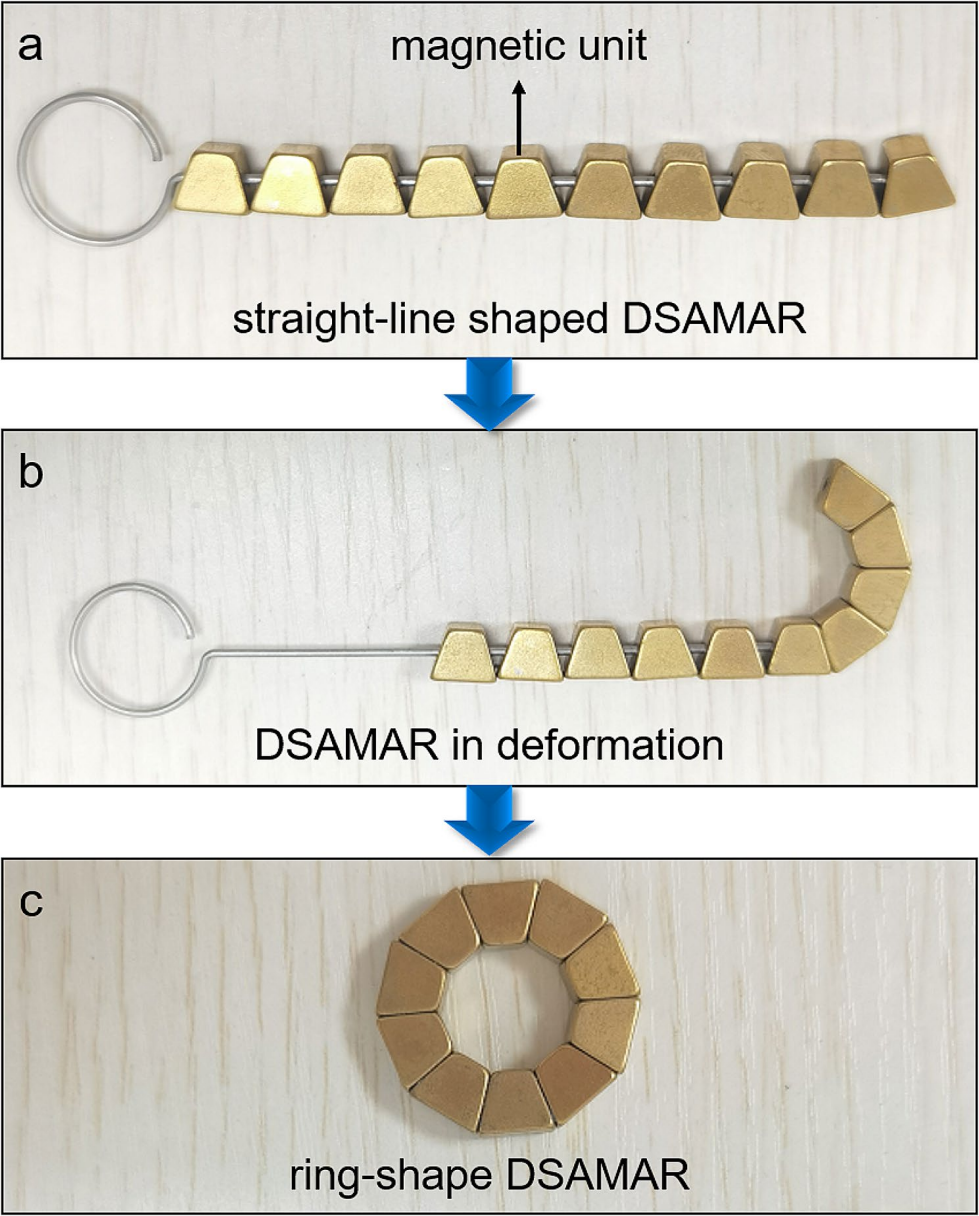
DSAMAR deformation process. a Straight-line-shaped DSAMAR. b DSAMAR in deformation. c Ring-shaped DSAMAR.

The deformable self-assembled magnetic fitting ring consists of 10 trapezoidal magnetic units machined from the NdFeb permanent magnet material and coated with titanium nitride on the surface. The mass of each magnetic unit was 1.25 g. The bevel of the magnetic unit had a central hole for the guide wire to pass through. The magnetic unit can be sequentially passed through the guide wire into a straight-line shape (Fig. 1a). When the magnetic units exit from the guide wire sequentially, the adjacent magnetic units can be attracted toward each other (Fig. 1b). When all magnetic units exit the guide wire, the straight-line-shaped magnetic units can be self-assembled into a ring shape (Fig. 1c). A complete DSAMAR has an outer diameter of 25 mm, an inner diameter of 13 mm, and a height of 5 mm. The magnetic force of two DSAMARs at zero distance is 184 N. Auxiliary devices include guides and catheters (Supplementary material 1). The DSAMAR self-assembly deformation process is shown in Supplementary material 2.

### Surgical procedures

**Fig. 2:**
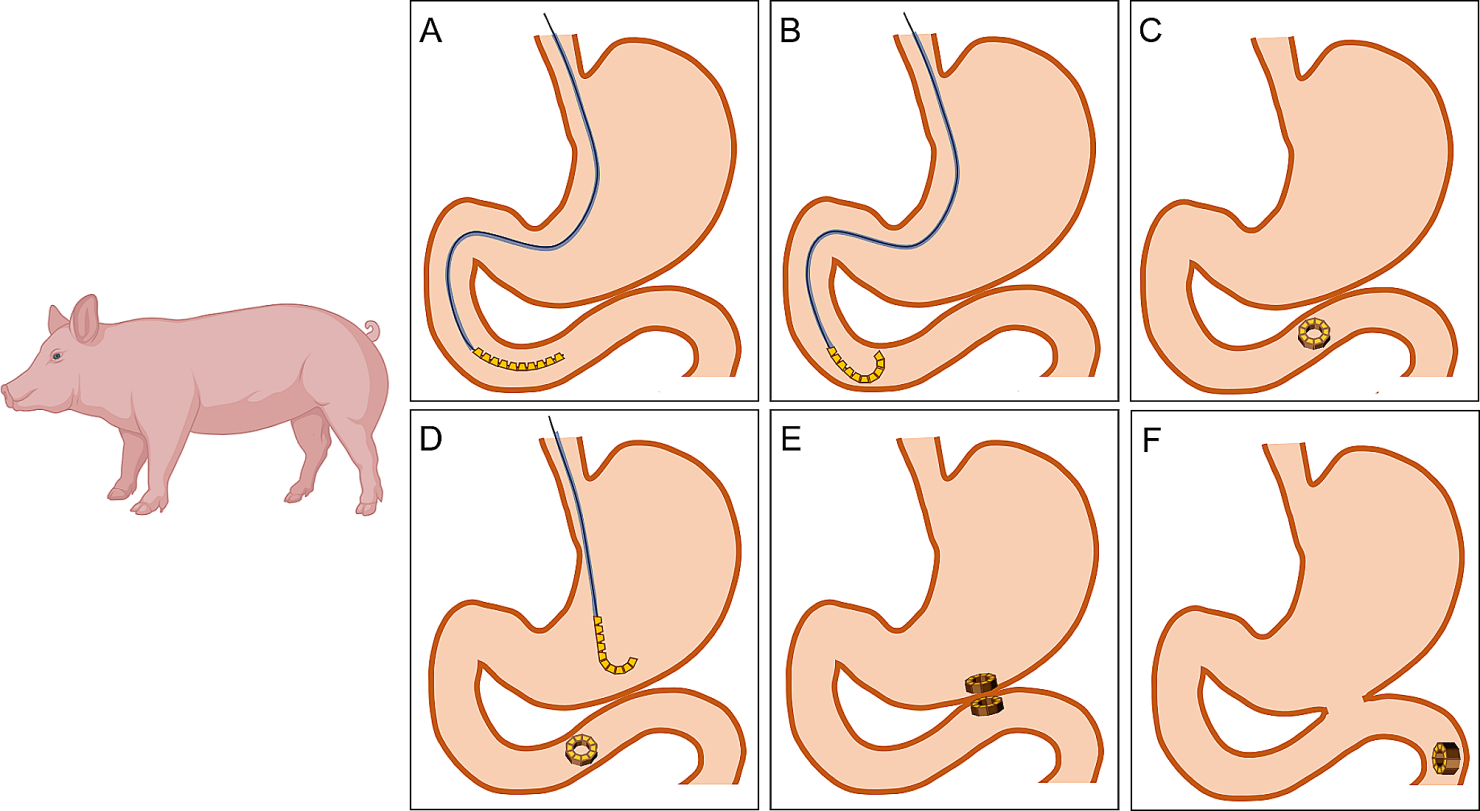
Surgical procedure. a Straight-line-shaped DSAMAR enters the duodenum. b DSAMAR is deformed in the duodenum. c DSAMAR becomes ring-shaped in the duodenum. d DSAMAR deformation process in the stomach. e The DSAMAR in the stomach is attracted to that at the end of the duodenum. f After the anastomosis is established, the DSAMARs enter the distal digestive tract

The experiment began with the adaptive feeding of the experimental pigs for 1 week after their purchase. They were fasted for 12 h before surgery. Pentobarbital sodium solution (30 mg/kg) was injected intravenously into their ears. After satisfactory anesthesia was achieved, the pigs were fixed on the operating table in the supine position and assisted by a tracheal intubation ventilator. The surgical procedure is shown in Fig. 2. The gastroscope was entered into the horizontal section of the duodenum through the mouth, and the guide wire head was placed at the end of the duodenum through the biopsy hole. The position of the guide wire head remained unchanged when the gastroscope was withdrawn. Next, 10 magnetic units were inserted into the body through the end of the guide wire. A push catheter was used to slowly push the magnetic unit into the body along the guide wire (Fig. 2a). The adjacent magnetic units were attracted toward each other and formed a ring (Fig. 2b, c). Next, the gastroscope and the guide wire were re-entered. The end of the guide wire was located in the stomach cavity. After removing the gastroscope, another 10 magnetic units were inserted through the end of the guide wire and the catheter was pushed into the stomach. The same method was used to make these 10 magnetic units in the stomach self-assemble into a ring (Fig. 2d). This DSAMAR in the stomach was attracted to the DSAMAR at the end of the duodenum (Fig. 2e). After establishing the gastrointestinal anastomosis, the DSAMARs entered the distal part of the digestive tract and eventually discharged through the anus (Fig. 2f).

### Postoperative care

After anesthesia, the pigs were fed the standard pig feed, paying special attention to their eating conditions and mental state. The DSAMAR-discharge time was recorded. Gastroscopy was performed to observe the anastomosis after the magnetic ring was discharged.

### Specimen collection and histological analysis

The experimental pigs were euthanized 4 weeks after the operation. All pigs were euthanized with excess barbital sodium (intravenous, 90 mg/kg pentobarbital sodium). Their whole stomach and duodenum were cut. Excess adipose tissues and connective tissues were removed. The healing of the anastomotic tissue was observed on the serosal surface. Next, the stomach and the duodenum were dissected to observe the healing of the anastomotic mucosal surface. All anastomosis-bearing segments with sufficient length on either side of the anastomosis were harvested. After gross observation, all samples were immersed overnight in 10% buffered formalin. After fixation, the samples were embedded in paraffin, and 4-µm-thick sections were cut at the anastomosis site. These sections were then stained with hematoxylin and eosin (H&E) and Masson dye for light microscopy.

### Statistical analysis

SPSS statistics software v20.0 was used for data analysis. Quantitative data were expressed as the mean ± standard deviation.

## Results

### Procedural parameters

**Fig. 3:**
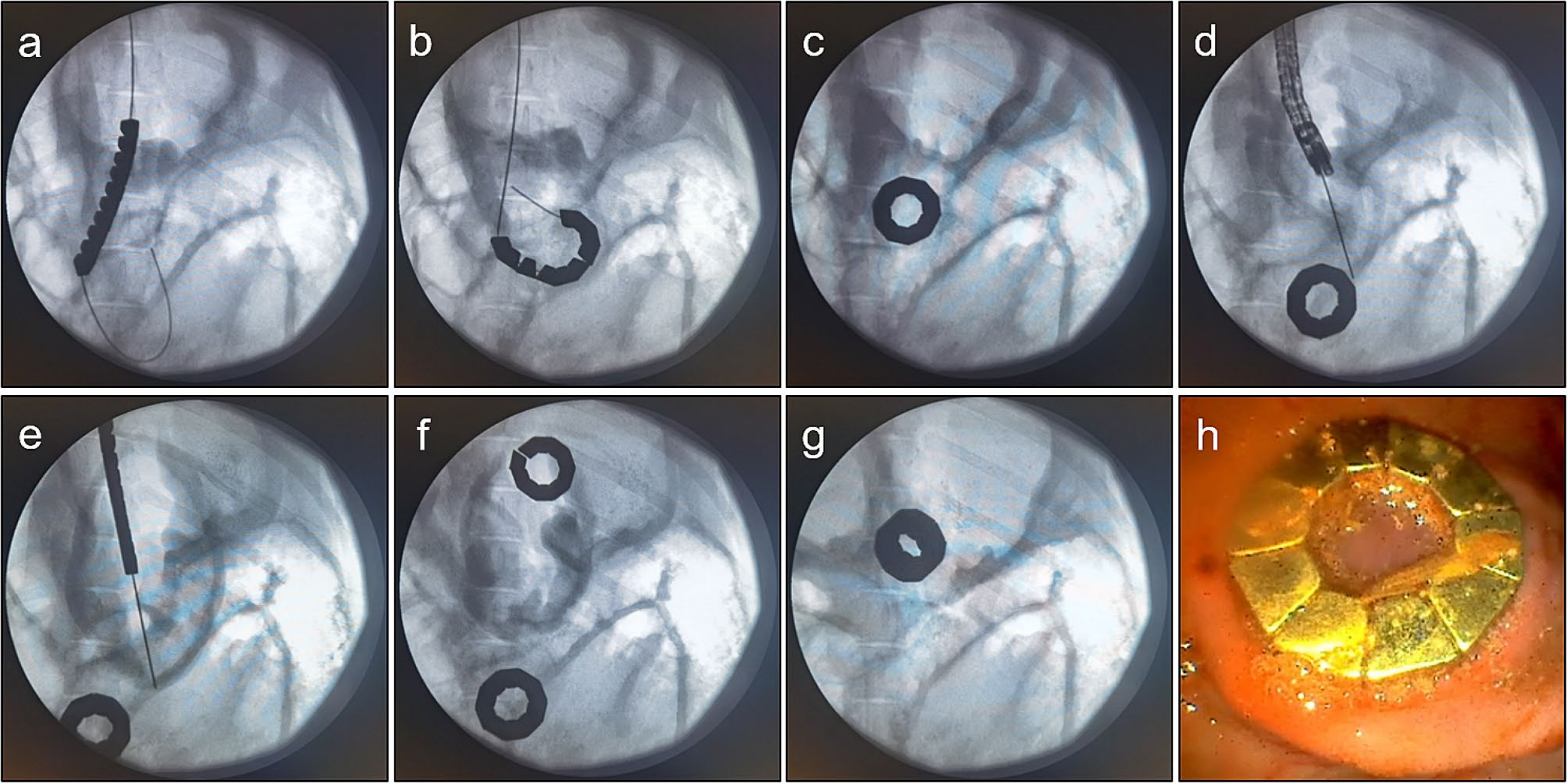
Animal experiments. a DSAMAR in the duodenum. b Deformation of the duodenal DSAMAR. c DSAMAR becomes circular in the duodenum. d A guide wire is inserted into the stomach with the aid of a gastroscope. e Linear DSAMAR enters the stomach. f DSAMAR becomes circular in the stomach. g X-ray showed that the two DSAMARs are attracted to each other. h DSAMAR in the stomach as seen by a gastroscope

The implantation process of the magnetic unit under the endoscope was facile. The linear DSAMAR successfully self-assembled into a ring. Gastrointestinal bypass magnetic anastomosis was successfully performed in all 10 pigs (Fig. 3a-f). No complications such as bleeding and perforation were observed. The whole endoscopy took 70.30 ± 19.05 min (range: 43–95 min) to complete. Intraoperative X-ray and gastroscopy showed good magnetic attraction (Fig. 3g, h).

### Survival rate and postoperative complications

All experimental pigs survived, resulting in a survival rate of 100% (10/10). The animals showed normal eating behavior and physical activities after the operation. The gastroscopy results showed that the gastroduodenal bypass magnetic anastomosis was smooth. No ulcer, erosion, or bleeding was observed in the anastomosis.

### Expulsion time of magnetic compression rings

**Fig. 4:**
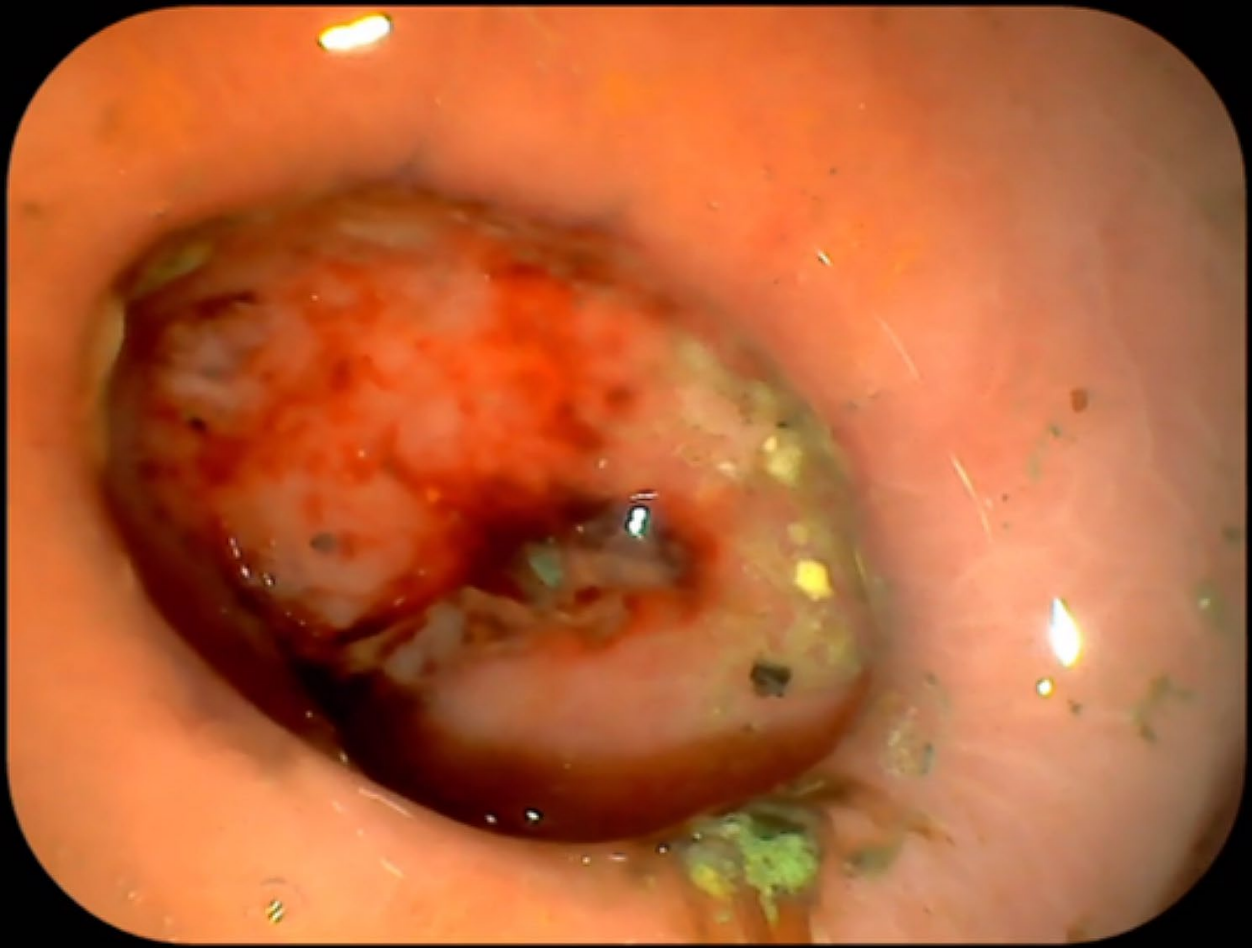
Gastroscopy showed that the gastrointestinal anastomosis was well formed

The mean time for defecating the DSAMARs was 13.60 ± 2.17 d (range: 10–17 d) after implantation. The data related to animal experiments are listed in Table 1. After the DSAMARs were discharged, the gastroscopy of the pigs showed that the gastrointestinal anastomosis was well formed (Fig. 4).

**Table 1:** Data related to animal experiments

Animal	Male/female	Weight (kg)	Operation time (min)	DSAMAR expelled time (d)	Device failure (Y/N)	Device-related adverse events (Y/N)
1	F	23	95	12	N	N
2	F	24.5	86	10	N	N
3	M	23	93	17	N	N
4	M	25	72	14	N	N
5	F	20	85	15	N	N
6	M	21.5	58	12	N	N
7	M	23	45	16	N	N
8	M	23.5	67	15	N	N
9	F	24	59	13	N	N
10	F	23	43	12	N	N

### Gross appearance of anastomosis

**Fig. 5:**
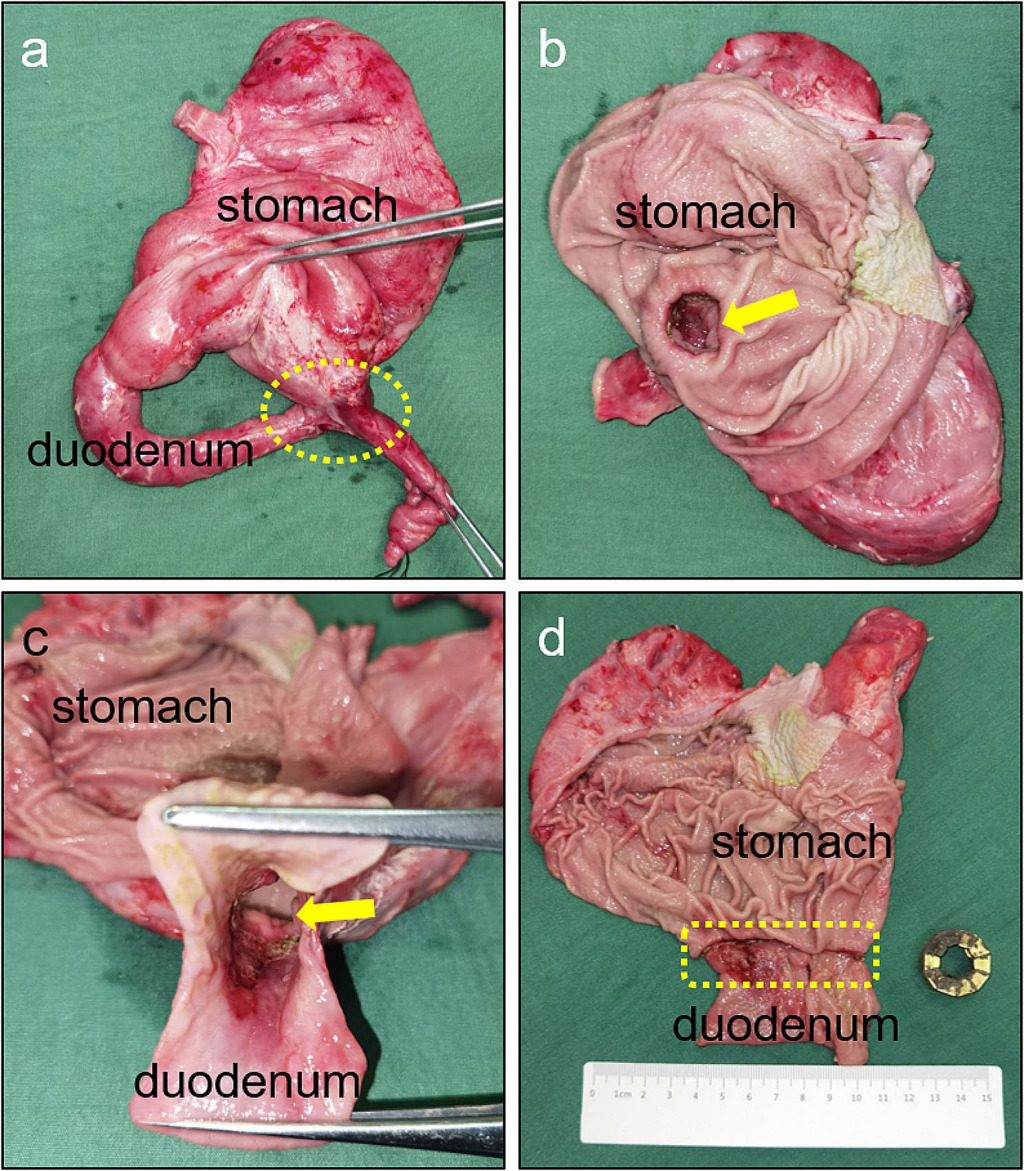
Gross specimen. a Gross specimen of gastrointestinal anastomosis. b The anastomosis is seen in the gastric cavity. c The anastomosis is seen in the intestinal cavity. d The anastomosis seen was dissected longitudinally

Four weeks after the surgery, the gross specimen of the anastomosis was obtained. The serous surface of the anastomosis healed well (Fig. 5a). The stomach and the small intestine showed good patency and the gastrointestinal anastomosis had healed well (Fig. 5b-d).

### Histological appearance of anastomosis

**Fig. 6:**
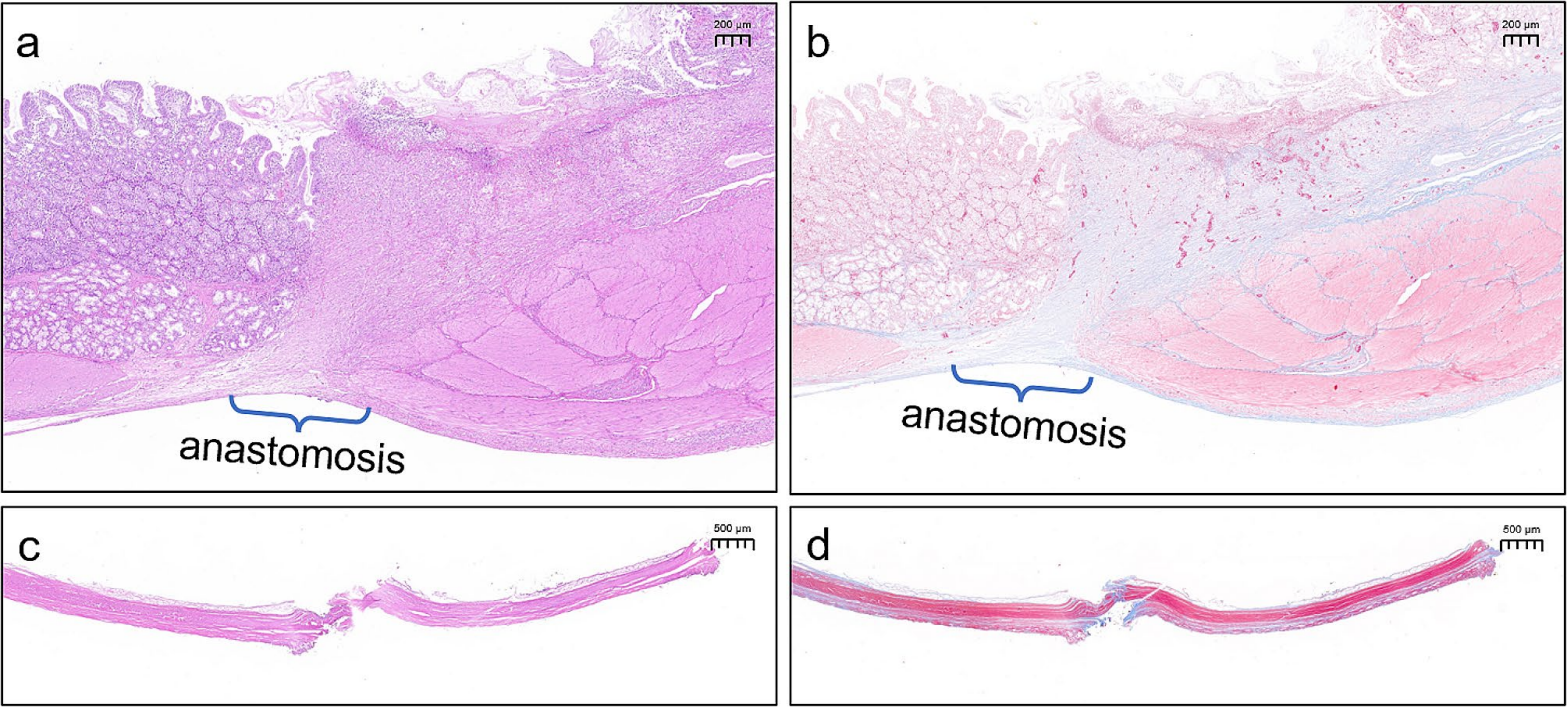
Histological specimen. a Hematoxylin and eosin staining of anastomosis. b Masson staining of anastomosis. c Hematoxylin and eosin staining of necrotic tissue. d Masson staining of necrotic tissue

H&E and Masson staining showed that the serous and mucosal layers of the anastomosis had healed well (Fig. 6a, b). The anastomotic area structure between the wall of the intestinal and the wall of the stomach was seen. The necrotic tissue between the magnets changed to segments, corresponding to the arrangement and distribution of each magnetic unit (Fig. 6c, d).

## Discussion

Magnetic compression anastomosis (MCA) is a novel anastomosis mode with widespread applications in cavity-organ anastomosis. It has been reported that MCA can be used in total digestive tract anastomosis [14–16], vascular anastomosis [17], ureterovesical anastomosis [18], cystostomy [19], rectovaginal fistula repair [20], and tracheoesophageal fistula repair [21]. MCA along with the interventional technique can be used for minimally invasive inferior portal vena cava shunt [22, 23]. The combined use of MCA and endoscopy in the digestive tract can be considered a new and superior model for digestive tract anastomosis.

MCA combined with endoscopic technique can be used for treating digestive tract stenosis. At present, this technique is mainly used for congenital esophageal atresia [24], acquired esophageal stenosis [25], and postoperative anastomotic stenosis of colorectal cancer [26], with good therapeutic effect. However, this technique is rarely used in gastric outflow tract obstruction, mainly because it is difficult to pass conventional magnets through the gastrointestinal stenosis. In addition, it requires highly flexible magnetic anastomosis devices. In 2011, Ryou designed Smart Self-Assembling MagnetS for ENdoscopy (SAMSEN) for application in endoscopic gastrojejunal anastomosis [11]. However, this magnet has only been explored in experimental animals. Later, Ryou designed a deformable magnet that could pass through the gastroscopic biopsy channel [27]. After passing the preclinical verification, it is now used mainly for performing endoscopic gastrointestinal bypass in obese patients [12]. However, no deformable magnetic anastomosis ring, especially for gastric outflow tract obstruction, has yet been designed.

The magnet used for endoscopic gastric outlet obstruction bypass anastomosis needs to meet two basic requirements. First, the magnet should be able to pass through the narrow part of the digestive tract. Second, the gastrointestinal bypass magnetic anastomosis should be large enough to ensure the smooth passage of food. The DSAMAR is a specially designed magnet for the endoscopic magnetic anastomosis of gastrointestinal stenosis. This magnet has the following characteristics: (1) It is arranged in a straight line through the narrow part of the digestive tract and self-assembles into a circle after reaching the anastomosis site. (2) Its deformation ratio (the ratio of the magnetic press area after forming a ring to the cross-sectional area when the line is in shape) is approximately 1:15, which allows large anastomosis with a magnet having a small cross-sectional area. (3) The self-assembly deformation process is simple. Although the placement process of DSAMAR requires the aid of an endoscope, the deformation process does not require an endoscope and can be completed only with a guide wire and a push tube, which greatly increases the flexibility of the application. (4) There are two main reasons for the long operation time in this study. First, this is an unprecedented operation method, the operator has not experienced repeated training, and the proficiency is low; Second, the stomach body of pigs is larger, and the difficulty of gastroscopy entering the duodenum is significantly higher than that of human operation.

Hence, the DSAMAR can be employed for endoscopic gastrointestinal bypass anastomosis. It has good applicability in implantation, deformation, and formation of anastomosis. Our study has several limitations too. First, only 10 experimental pigs were used, which resulted in a small sample size. Second, normal experimental pigs were used in this study instead of those with gastric outflow tract obstruction. However, we believe that these shortcomings do not affect the evaluation of the flexibility and compatibility effect of DSAMARs. Patients were unable to eat orally during the period before the establishment of magnetic anastomosis, which was a limitation of magnetic anastomosis compared with EUS-guided gastrojejunostomy.

In conclusion, the DSAMARs were found to be a safe and feasible tool for endoscopic gastrointestinal bypass anastomosis, making them potentially clinically applicable.

### Electronic supplementary material

Below is the link to the electronic supplementary material.

Supplementary Material 1

Supplementary Material 2

## Data Availability

The datasets generated and/or analyzed in the experiment are available from the corresponding author on reasonable request.

## Abbreviations

DSAMAR: Deformable self-assembled magnetic anastomosis rings
MGOO: Malignant gastric outlet obstruction
MCA: Magnetic compression anastomosis
SAMSEN: Smart Self-Assembling MagnetS for Endoscopy
