# Supplementary figures and images for: Endoscopic gastrointestinal bypass anastomosis using deformable self-assembled magnetic anastomosis rings (DSAMARs) in a pig model
Source: BMC Gastroenterol. 2024 Jan 5;24:20. doi: 10.1186/s12876-024-03122-0 (PMC10768203; doi:10.1186/s12876-024-03122-0)

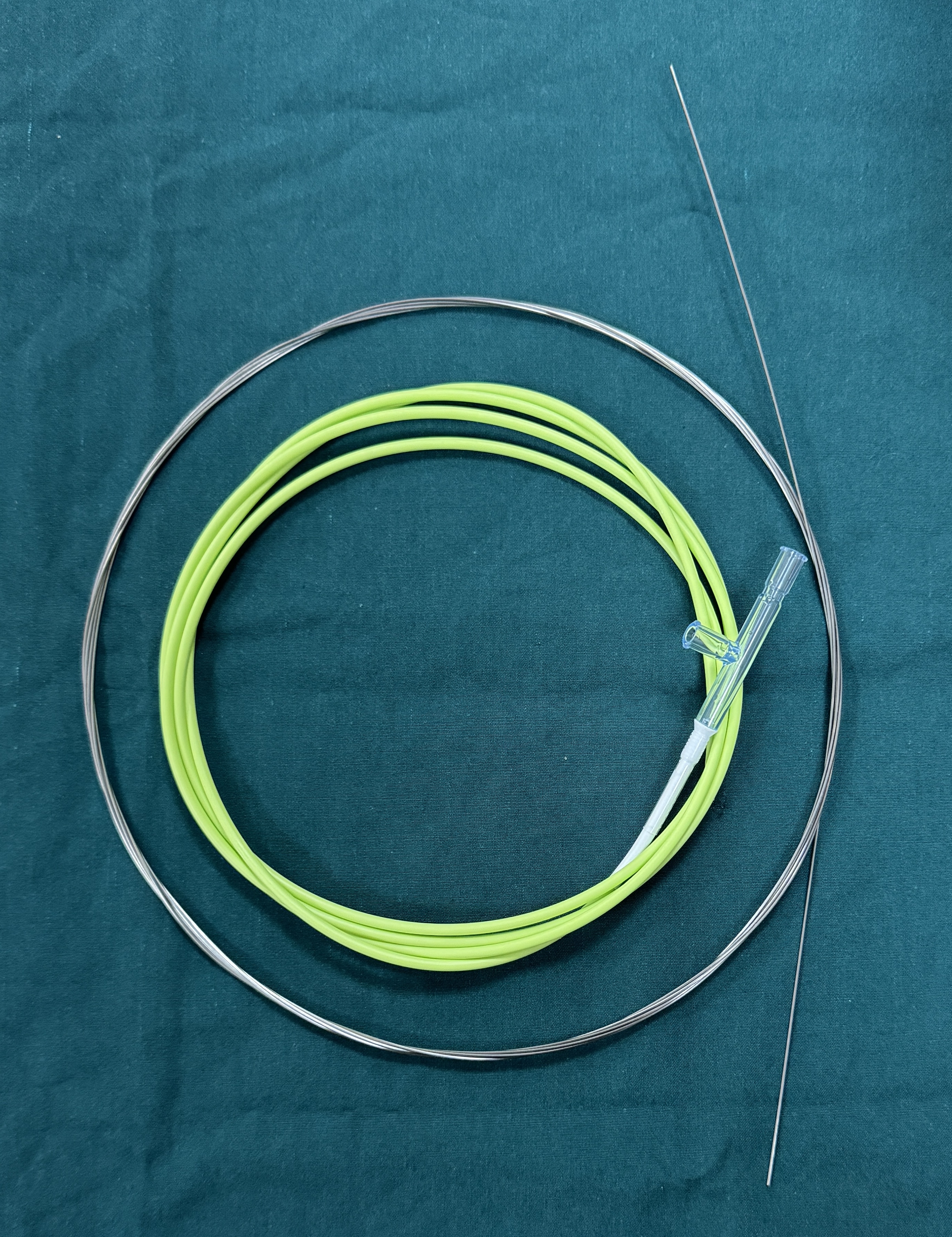

Supplement: Supplementary file 1 — Supplementary Material 1 [file 12876_2024_3122_MOESM1_ESM.jpg]
